# Hypersensitivity reactions to dengue vaccine TAK-003 (Qdenga®) following an immunization campaign targeting children and adolescents

**DOI:** 10.1590/0037-8682-0198-2026

**Published:** 2026-06-26

**Authors:** Márcia Paulliny Soares Bahia, Silon de Souza Gomes, Argus Leão Araújo, Hoberdan Oliveira Pereira, José Geraldo Leite Ribeiro, Alexandre Sampaio Moura

**Affiliations:** 1 Prefeitura de Belo Horizonte, Gerência de Vigilância, Belo Horizonte, MG, Brasil.; 2 Hospital Eduardo de Menezes, Departamento de Infectologia, Belo Horizonte, MG, Brasil.; 3 Faculdade de Ciências Médicas de Minas Gerais, Curso de Medicina, Belo Horizonte, MG, Brasil.; 4 Faculdade de Saúde Santa Casa de Belo Horizonte, Programa de Pós-Graduação Stricto Sensu, Belo Horizonte, MG, Brasil.

**Keywords:** Dengue, Dengue vaccine, Hypersensitivity, Anaphylaxis, Adverse Effects

## Abstract

**Background:**

A national dengue vaccination campaign using TAK-003 was launched in 2024, targeting adolescents. Post-licensure surveillance suggested higher-than-expected hypersensitivity reactions.

**Methods:**

We conducted a descriptive study using vaccine adverse event data and vaccination records from Belo Horizonte among individuals aged 6-14 years.

**Results:**

Of the 146,115 doses administered, 28 hypersensitivity reactions were reported (191.6 per million), including nine cases of anaphylaxis and two of anaphylactic shock. All events followed the first dose. Mean age was 9.4 years, and mean time to symptom onset was 31 minutes.

**Conclusions:**

Hypersensitivity rates exceeded expected vaccine-associated levels, reinforcing the need for continued pharmacovigilance.

TAK-003 (Qdenga®), a tetravalent dengue vaccine developed by Takeda, was approved in Brazil in 2023. The national public immunization campaign began in February 2024 and targeted individuals aged 10 to 14 years^1^. 

The efficacy and safety of TAK-003 in this age group have been previously demonstrated in large clinical trials, with surveillance data for up to 5 years post-vaccination^2^ ^-^ ^4^. Safety analysis of these clinical trials showed similar incidence of adverse events in vaccine and placebo groups^2^. Furthermore, the evaluation of long-term (4-5 years) vaccine safety did not identify any cases of anaphylaxis^4^. A meta-analysis focusing on the immunogenicity, efficacy, and safety of the TAK-003 vaccine reported two cases classified as hypersensitivity reactions (one in the placebo group)^5^. However, during the large-scale vaccination program in Brazil, a considerable number of cases of anaphylaxis and other hypersensitivity reactions were reported. In the first post-licensure pharmacovigilance study published by the Ministry of Health of Brazil in October 2024, the overall rate of anaphylaxis or anaphylactic shock was 63.1 per 1,000,000 vaccine doses administered^6^. The observed rate exceeds previously reported rates of anaphylaxis as an adverse event following immunization (AEFI), which range from 1 per 100,000 to 1 per 1,000,000 vaccine doses administered^7^. 

This study aimed to describe the frequency of anaphylaxis and hypersensitivity reactions following TAK-003 vaccination in Belo Horizonte from February 2024 to February 2025. Belo Horizonte has a population of 2.3 million people and has experienced several dengue outbreaks since 1998. In 2024, Belo Horizonte experienced a major dengue outbreak, with 218,539 confirmed cases, of which 44,686 occurred among children aged 5 to 19 years. The target population for the dengue vaccination campaign in Belo Horizonte initially consisted of children aged 10 to 14 years. This age range gradually expanded to include children aged 6 to 14 years. 

Data on vaccine adverse events occurring in children aged 6 to 14 years who were vaccinated with TAK-003 in primary care clinics during the study period were retrieved from the National Vaccine Adverse Event Reporting System (e-SUS Notifica). Initially, all reported AEFIs related to the dengue vaccine were extracted. Subsequently, an infectious disease medical expert conducted a comprehensive manual review of all reports, and cases describing allergic reactions were selected. To ensure a systematic search, MedDRA (Medical Dictionary for Regulatory Activities) terms were also used, including anaphylaxis, anaphylactic shock, urticaria, itching, angioedema, hypersensitivity, and allergy.

Cases of Type 1 hypersensitivity reactions were defined as those presenting with at least one symptom of cutaneous or systemic involvement suggestive of an allergic reaction, such as urticaria, erythema, pruritus, angioedema, cough, sneezing, runny nose, dyspnea, respiratory distress, or other symptoms. Based on the analysis of the signs and symptoms presented, these cases were classified according to the Brighton criteria to assess the level of certainty regarding anaphylaxis. Level 1 certainty was defined as sudden onset and rapid progression of signs and symptoms, with ≥1 major skin reaction and ≥1 major respiratory and/or cardiac reaction^7^. Data on the number of vaccine doses administered in public primary care clinics were retrieved from the National Immunization Program Information System.

Categorical variables are presented as frequencies and proportions, and continuous variables are presented as means and standard deviations. Event rates were calculated using the total number of doses administered in the public health system as the denominator and the total number of each event as the numerator. The study was approved by the Ethics Committee of the Prefeitura de Belo Horizonte (CAAE #89758925.6.0000.5140) and was conducted in accordance with the ethical standards of the Institutional Human Experimentation Committee and the Declaration of Helsinki.

From February 2024 to February 2025, 146,115 doses were administered to individuals aged 6-14 years in Belo Horizonte, of which 104,286 were first doses, and 41,829 were second doses. All patients in the study were vaccinated in a public primary care clinic.

During the same period, 28 AEFIs classified as hypersensitivity reactions were reported to the *e-SUS Notifica* system, corresponding to an incidence rate of 191.6 per million doses administered. However, all cases of hypersensitivity reactions occurred after the first dose, resulting in an incidence rate of 268.4 per million first doses. Hypersensitivity reactions were associated with eight different vaccine batches. 

Of the total reported cases, 12 (42.8%) were male, and the median age was 9.39 years (range: 6-14 years) (Table 1). None of the patients had TAK-003 co-administered with other vaccines. Only five (17.2%) had a history of allergic reactions (two to seafood, one to amoxicillin, and two unspecified). The mean time between vaccine administration and the onset of signs and symptoms was 31.2 ± 28.8 minutes, with 12 (42.8%) occurring within 15 minutes, 7 (25.0%) between 15 and 30 minutes, and 9 (32.1%) after 30 minutes (Table 1).

**TABLE 1:** Sociodemographic and clinical characteristics of patients experiencing hypersensitivity reactions following TAK-003 vaccination (n = 28).

Characteristic	Value
Male - n (%)	12 (42.9)
Age (years) - median (range)	9.4 (6-14)
Time between vaccination and event - n (%)	
≤15 minutes	12 (42.9)
16‒30 minutes	7 (25.0)
>30 minutes	9 (32.1)
Brighton classification - n (%)	
Level 1	9 (32.1)
Level 2	0 (0)
Level 3	2 (7.1)
Level 4	2 (7.1)
Level 5	15 (53.3%)

Most individuals (96.4%) received medical care, and 92.4% received prescribed medications. Epinephrine was administered in three cases (10.7%). All individuals fully recovered and were discharged within 24 hours of observation at the health facility. In 13 (46.4%) individuals, a subsequent dose of the TAK-003 vaccine was contraindicated. None of the 15 patients for whom a second dose was permitted with caution received it.

Of the 28 cases of hypersensitivity reactions, 9 (32.1%) were classified as anaphylaxis with Brighton diagnostic certainty level of 1 (incidence rate: 191.6 [95% confidence interval (CI), 127.3-277.0] per million doses and 61.6 [95% CI 28.2-116.9] per million doses, respectively). A detailed description of the sociodemographic characteristics and symptoms presented by patients with confirmed anaphylaxis is provided in Table 2.

**TABLE 2:** Major symptoms according to the Brighton classification among cases classified as anaphylaxis (n = 9).

Case	Sex (M/F)	Age (years)	Major symptoms
Patient 1	M	11	Urticaria and signs of respiratory distress
Patient 2	F	13	Urticaria and signs of respiratory distress
Patient 3	F	11	Angioedema, signs of respiratory distress, and loss of consciousness (non-vasovagal)
Patient 4	F	10	Angioedema involving the upper airways, generalized erythema, and itching
Patient 5	M	14	Generalized erythema, itching, and signs of respiratory distress
Patient 6	M	9	Generalized erythema and itching, expiratory wheeze, and signs of respiratory distress
Patient 7	M	10	Angioedema, signs of respiratory distress, and hypotension
Patient 8	F	6	Angioedema and signs of respiratory distress
Patient 9	F	9	Urticaria, angioedema, signs of respiratory distress, and hypotension

Two cases (7.1%) were diagnosed with anaphylactic shock. One case occurred in a 10-year-old male who presented with dyspnea, dry cough, facial edema, and hypotension 15 minutes after receiving the first vaccine dose. He was initially managed with intravenous fluids, intramuscular (IM) epinephrine, and salbutamol, and was referred to inpatient care. The second case involved a 9-year-old female who developed angioedema, generalized urticaria, coughing, hypotension, and dyspnea with hypoxemia 30 minutes after receiving the first vaccine dose. Oxygen supplementation, IM epinephrine, and salbutamol were promptly administered, followed by referral to an emergency unit.

From the beginning of the dengue vaccination campaign with TAK-003 through February 2025, hypersensitivity-associated AEFI rates in Belo Horizonte were high. The safety of TAK-003 in children has been well established in large clinical trials conducted by Takeda. Long-term follow-up data from a phase 3 trial revealed no serious adverse events considered related to the investigational product and no reports of hypersensitivity or anaphylaxis^4^.

Between March 2023 and September 2024, a total of 501 hypersensitivity reactions associated with the TAK-003 vaccine were reported across Brazil, corresponding to a reporting rate of 147.1 events per 1,000,000 administered doses. Among these cases, 124 were classified as anaphylaxis, representing a rate of 36.4 per 1,000,000 doses^8^. In a mass immunization campaign in Dourados, Brazil, the estimated rate of anaphylaxis was 16.0 (4.4-58.5) per million doses^9^. In Argentina, passive surveillance of TAK-003 AEFIs has revealed similar rates of hypersensitivity reactions (140 per 1,000,000 doses) but lower rates of anaphylaxis (6 per 1,000,000 doses)^10^. 

In Belo Horizonte, we observed a higher rate of hypersensitivity reactions (191.6 per 1,000,000 doses), including anaphylaxis (61.6 per 1,000,000 doses). Vaccine quality-related issues are unlikely to account for the higher hypersensitivity rates, as events were observed across multiple vaccine batches. Hypersensitivity reactions occurred across different ages and both sexes, indicating no specific pattern. 

The underreporting of administered doses does not explain the observed higher rates of hypersensitivity reactions. In Belo Horizonte, the SIPNI data are directly obtained from the electronic health records of the primary healthcare units where vaccines are administered. Notably, there were no school-based vaccination campaigns during the study period. The risk of a spurious finding due to AEFI misclassification was also mitigated by applying well-established definitions.

Since the initial reports of anaphylactic reactions to TAK-003 in Brazil, the Ministry of Health has taken a series of actions, primarily focused on assessing vaccine quality. The National Institute for Quality Control in Health (INCQS) investigated the presence of endotoxins in the implicated vaccine batches. The results showed no detectable endotoxins, effectively ruling out bacterial contamination during manufacturing or storage^8^. In collaboration with other institutions, the Ministry of Health is supporting studies to assess factors associated with anaphylaxis and to identify specific allergens and the underlying mechanisms of hypersensitivity reactions^8^.

The limitations of this study need to be acknowledged. First, this study was based on passive surveillance, which depends on healthcare professionals' awareness of the importance of reporting AEFIs. Therefore, although we identified a higher rate of hypersensitivity reactions, the number of cases, particularly those involving mild reactions, may still be underestimated. In addition, the study was conducted in a single region; therefore, further studies including data from other Brazilian regions are needed. Finally, misclassification of hypersensitivity reactions cannot be excluded. Although we used Brighton criteria to mitigate this risk, accurate classification depends on the quality and completeness of data recorded in the *e-SUS Notifica* system, which is filled out by different healthcare providers.

In summary, the incidence of hypersensitivity reactions associated with the TAK-003 vaccine during an immunization campaign among children and adolescents was higher than expected. Additional studies are warranted to elucidate the underlying etiology of these reactions and further characterize the safety profile of the vaccine. 

## Data Availability

Data available upon request.
